# Association of high sensitivity C-reactive protein-triglyceride glucose index and chronic kidney disease: a cross-sectional study

**DOI:** 10.3389/fendo.2026.1781798

**Published:** 2026-02-13

**Authors:** Fengxu Zhang, Zhengfang Wang, Han Zhang

**Affiliations:** Health Management Center, Beijing Aerospace General Hospital, Beijing, China

**Keywords:** CKD, Cross-sectional study, cti, Hs-CRP, insulin resistance

## Abstract

**Background:**

Chronic kidney disease (CKD) represents a significant global health burden. Its pathogenesis is closely linked to a state of metabolic inflammation, involving insulin resistance and chronic low-grade inflammation. The high-sensitivity C-reactive protein-triglyceride glucose index (CTI) is a novel composite biomarker integrating inflammatory and metabolic information, yet its association with CKD in the general population remains unclear.

**Objective:**

This study aimed to investigate the cross-sectional association between the CTI and the prevalence of CKD in a health examination population.

**Methods:**

A total of 10–287 adults who underwent routine health check-ups were included. The CTI was calculated as the product of high-sensitivity C-reactive protein (hs-CRP) and the triglyceride-glucose (TyG) index. CKD was defined as an estimated glomerular filtration rate (eGFR) <60 mL/min/1.73 m². Logistic regression models were used to assess the association of CTI (as a continuous variable and in quartiles) with CKD. Diagnostic performance was evaluated using the area under the receiver operating characteristic curve (AUC). The shape of the association and its heterogeneity were explored using restricted cubic splines and subgroup analyses.

**Results:**

A total of 163 (1.58%) participants were identified as CKD. After full adjustment for confounders, each unit increase in CTI was associated with a 2.25-fold increased odds of CKD (OR = 2.25, 95%CI: 1.66–3.06). Compared to participants in the lowest CTI quartile, those in the highest quartile had a 2.17-fold higher risk of CKD (OR = 2.17, 95% CI: 1.25–3.76; *P* for trend = 0.001). A nonlinear dose-response relationship was observed between CTI and CKD (*P* for nonlinearity = 0.012). The CTI demonstrated superior diagnostic performance for CKD (AUC = 0.69, 95% CI: 0.64–0.73) compared to its individual components, the TyG index (AUC = 0.66) and hs-CRP (AUC = 0.59). Subgroup analyses revealed that the association was particularly pronounced in males, individuals aged <60 years, and those with a history of alcohol consumption.

**Conclusion:**

In a health examination population, a higher CTI level was independently and nonlinearly associated with an increased prevalence of CKD, and it showed better diagnostic performance than individual inflammatory or metabolic markers alone. The CTI may serve as a useful tool for identifying individuals at high risk for CKD, especially for early risk stratification in specific subgroups.

## 1 Introduction

Chronic kidney disease (CKD) constitutes a major and growing global public health challenge. It currently affects over 800 million individuals worldwide, and its associated disease burden has risen sharply over the past thirty years, solidifying its role as a powerful, independent risk factor for cardiovascular disease, kidney failure, and all-cause mortality (1–3). A critical obstacle in addressing this burden is the frequently asymptomatic nature of early-stage CKD, which leaves a vast number of individuals undiagnosed until substantial, often irreversible, organ damage has occurred, thereby missing the optimal window for intervention (4, 5). Consequently, the identification of novel, simple, and effective biomarkers for the early detection of renal risk in the general and at-risk populations is paramount for improving both primary and secondary prevention.

The pathogenesis of CKD is multifactorial, involving a complex interplay of genetic, metabolic, hemodynamic, and inflammatory pathways. Central to this complexity is the concept of “metabolic inflammation” (meta-inflammation), which has emerged as a key pathophysiological link connecting obesity, insulin resistance, and the development of CKD (6, 7). Insulin resistance contributes to renal injury through mechanisms such as glomerular hyperfiltration, activation of the intrarenal renin-angiotensin-aldosterone system, and promotion of ectopic lipid deposition (8). Concurrently, chronic low-grade inflammation drives progressive kidney damage via the sustained release of pro-inflammatory cytokines, leading to endothelial dysfunction, oxidative stress, and fibrosis (9). These pathways do not operate in isolation; they engage in a vicious, self-reinforcing cycle that synergistically accelerates renal injury. This synergy suggests that an integrated assessment of both metabolic and inflammatory status may offer a more comprehensive evaluation of renal risk than either dimension alone.

In clinical and research settings, the triglyceride-glucose (TyG) index is widely validated as a reliable and practical surrogate marker of insulin resistance, with numerous studies confirming its association with CKD (10, 11). Similarly, high-sensitivity C-reactive protein (hs-CRP), a classic marker of systemic inflammation, has been consistently linked to the onset and progression of CKD (12–14). However, employing these markers in isolation may fail to fully capture the integrated burden of the synergistic “metabolic inflammation” state. Theoretically, a composite index that combines both dimensions could provide a more holistic quantification of this pathological load and potentially yield superior predictive power. To this end, we propose the High-sensitivity C-reactive protein-Triglyceride-glucose Index (CTI), defined as the product of hs-CRP and the TyG index. This novel metric is specifically designed to directly reflect the multiplicative interaction between chronic inflammation and insulin resistance.

While research into other composite inflammatory-metabolic indices is emerging, epidemiological evidence specifically concerning the CTI, particularly derived from large-scale, general health-examination populations, remains scarce (15–17). This gap limits our understanding of its utility in broad, community-based screening contexts.

Therefore, utilizing a large-scale health examination cohort, this cross-sectional study aimed to systematically investigate the association between the CTI and the presence of CKD. We hypothesized that a higher CTI would be independently associated with an increased prevalence of CKD. By validating the CTI as a novel integrative biomarker, this study seeks to provide methodological insights for early risk stratification and inform future clinical screening practices.

## 2 Methods

### 2.1 Study design and participants

A retrospective, single-center, cross-sectional study was conducted utilizing data from the health examination center of Beijing Aerospace General Hospital. Adults (aged ≥18 years) who underwent comprehensive health check-ups between January 1, 2024, and December 31, 2024. Participant data were obtained directly from the hospital’s standardized electronic health record system, which integrates laboratory results, vital signs, and questionnaire responses. The initial dataset comprised 12–976 individuals. Data processing and quality control involved the following steps: First, We applied the following exclusion criteria: (1) missing data for key variables required to calculate the CTI (hs-CRP, fasting plasma glucose [FPG], fasting triglycerides [TG]) or to define CKD (serum creatinine) (n=1 824); (2) self-reported history of renal replacement therapy (dialysis or kidney transplantation) (n=52); (3) potential acute conditions that could significantly distort inflammatory markers, defined as a white blood cell count >10×10^9^/L or a recorded body temperature >37.5 °C on the examination day (n=813). Laboratory measurements were performed following standardized clinical protocols, with all assays subject to the laboratory’s internal quality control procedures. After exclusions, a total of 10–287 participants were included in the final analysis. As this was a retrospective cross-sectional study utilizing an existing database, the sample size was determined by data availability and feasibility. All eligible participants meeting the inclusion criteria within the study period were analyzed. Prior to statistical modeling, continuous variables were assessed for implausible extreme values, and logical checks were performed on categorical data. The study protocol was reviewed and approved by the Institutional Review Board of Beijing Aerospace General Hospital [Approval No: (2025)clinical(25)], which waived the requirement for informed consent due to the retrospective and anonymized nature of the data analysis.

### 2.2 Data collection and variable definitions

All participants underwent a standardized protocol, including a structured questionnaire, physical examinations, and collection of fasting venous blood.

All laboratory measurements were performed in the central laboratory of Beijing Aerospace General Hospital, which follows standardized operating procedures. Fasting blood samples were collected and analyzed using automated clinical chemistry analyzers. Specifically, complete blood count (including white blood cell count for exclusion criteria) was performed on a Sysmex XN-10(B4) automated hematology analyzer using the manufacturer’s specific diluent and lysing reagents. Biochemical analyses, including the measurement of hs-CRP, fasting plasma glucose, fasting triglycerides, and serum creatinine, were conducted on a Toshiba TBA-FX8 automated biochemical analyzer. hs-CRP was quantified using a particle-enhanced immunoturbidimetric assay. Fasting plasma glucose was measured by the hexokinase method, and fasting triglycerides were determined using an enzymatic colorimetric method (GPO-PAP). Urinalysis for protein was performed using an AVE-752 urine analyzer. All assays were calibrated according to the manufacturer’s protocols using traceable standards. Internal quality control was performed daily using commercial control materials, including the Sysmex XN CHECK for hematology and appropriate biochemical controls. Furthermore, the laboratory participates in the National Center for Clinical Laboratories proficiency testing scheme to ensure the accuracy and reproducibility of all measurements.

Exposure Variable: The CTI was calculated as follows: CTI = 0.412× Ln (hs-CRP [mg/L[) + Ln (TG [mg/dL] × FPG [mg/dL])/2 (18). The TyG Index was computed using the formula: TyG Index = Ln [Fasting TG (mg/dL) × FPG (mg/dL)/2] (19). Serum hs-CRP was measured using a particle-enhanced immunoturbidimetric assay.

Outcome Variable: CKD was defined according to the Kidney Disease: Improving Global Outcomes (KDIGO) 2012 guidelines as either an estimated glomerular filtration rate (eGFR) < 60 mL/min/1.73 m² (20).

eGFR was calculated using the Chronic Kidney Disease Epidemiology Collaboration (CKD-EPI) equation without the race coefficient (21).

Covariates: The following potential confounding variables were collected: age (years), sex (male/female), body mass index (BMI, kg/m²), systolic and diastolic blood pressure (SBP, DBP, mmHg), current smoking status (yes/no), current alcohol consumption (yes/no), history of diabetes (self-reported or FPG ≥7.0 mmol/L), history of hypertension (self-reported or SBP/DBP ≥140/90 mmHg), serum total cholesterol (TC), high-density lipoprotein cholesterol (HDL-C), low-density lipoprotein cholesterol (LDL-C) levels.

### 2.3 Statistical analysis

Statistical analyses were performed using SPSS 25.0 and R 4.1.3 software. A two-sided *P* < 0.05 was considered statistically significant. Measurement data were presented as the mean ± standard deviation. Normally distributed data were analyzed using Student’s t-tests. Non-normally distributed data were analyzed using a Wilcoxon rank sum test. Logistic regression analysis were conducted to estimate OR and 95%CI for the associations of the CTI with CKD. CTI was analyzed in two forms: (a) as a continuous variable (per 1-unit increase) and (b) as a categorical variable (divided into quartiles Q1-Q4, with Q1 as the reference). Three nested models were constructed: Model 1 (unadjusted), Model 2 (adjusted for age, gender, BMI), Model 3 (further adjusted for hypertension, diabetes, smoking, drinking). Test for linear trend across increasing CTI quartiles was performed by treating the quartile variable as an ordinal term in the regression model. Restricted cubic splines (RCS) with three knots were applied to evaluate potential nonlinear associations between exposure variables and CKD risk. Subgroup analyses were performed by stratifying participants by age (<60 vs. ≥60 years), gender, smoking, drinking, presence of diabetes, and presence of hypertension. Covariates were selected based on their established status as major risk factors for CKD and their potential to confound the association between metabolic-inflammatory markers and kidney function.

## 3 Results

### 3.1 Baseline characteristics of the participants

A total of 10–287 participants were included in the analysis. 163 (1.58%) individuals were identified as CKD, while 10 124 (98.42%) comprised the non-CKD group. The baseline characteristics of the participants and comparisons between groups were detailed in **Table 1**. Participants with CKD were significantly older than those without CKD (55.68 ± 15.5 vs. 45.76 ± 13.64 years, *P* < 0.001). The proportion of males was comparable between the two groups (69.3% vs. 65.0%, *P* = 0.254). Regarding metabolic and inflammatory profiles, the CKD group exhibited significantly higher levels of BMI, hs-CRP, FBG, TG, and the TyG (all *P* < 0.001). Consequently, the composite CTI was markedly elevated in the CKD group compared to the non-CKD group (1.54 ± 0.29 vs. 1.16 ± 0.18, *P* < 0.001). Furthermore, traditional cardiovascular and renal risk factors were more prevalent among CKD patients, including higher systolic and diastolic blood pressure and a greater proportion of individuals with a history of hypertension and diabetes (all *P* < 0.001). No significant differences were observed between groups for LDL-C or the prevalence of alcohol consumption.

**Table 1:** Basic characteristics of cohort study participants (N = 10287).

Group	Total (N = 10287)	CKD (n=163)	Non-CKD (n=10124)	*P*
Age (years)	45.91 ± 13.73	55.68 ± 15.5	45.76 ± 13.64	<0.001
Male (n,%)	6697 (65.1)	113 (69.3)	6584 (65.0)	0.254
BMI (kg/m^2^)	25.39 ± 3.83	27.02 ± 4.73	25.36 ± 3.81	<0.001
hs-CRP	2.23 ± 2.9	3.22 ± 3.77	2.21 ± 2.89	<0.001
FBG (mmol/L)	5.51 ± 1.19	6.82 ± 2.78	5.49 ± 1.13	<0.001
TC (mmol/L)	5.06 ± 0.97	5.26 ± 1.11	5.05 ± 0.97	0.006
TG (mmol/L)	1.75 ± 1.17	2.41 ± 1.75	1.74 ± 1.15	<0.001
LDL-C (mmol/L)	2.74 ± 0.73	2.79 ± 0.81	2.74 ± 0.73	0.346
HDL-C (mmol/L)	1.22 ± 0.28	1.17 ± 0.31	1.22 ± 0.28	0.021
TyG	1.41 ± 0.58	1.86 ± 0.78	1.4 ± 0.57	<0.001
CTI	1.17 ± 0.26	1.54 ± 0.29	1.16 ± 0.18	<0.001
Smoking (n, %)	2126 (20.7)	46 (28.2)	2080 (20.5)	0.017
Drinking (n, %)	4198 (40.8)	59 (36.2)	4139 (40.9)	0.222
SBP (mmHg)	126 ± 18	141 ± 23	125 ± 18	<0.001
DBP (mmHg)	75 ± 11	82 ± 15	75 ± 11	<0.001
Hypertension(n, %)	1818 (17.7)	86 (52.8)	1732 (17.1)	<0.001
Diabetes(n, %)	741 (7.2)	42 (25.8)	699 (6.9)	<0.001

BMI, body mass index; FBG, fasting blood glucose; TC, cholesterol; TG, triglycerides; LDL-C, low density lipoprotein-cholesterol; HDL-C, high density lipoprotein-cholesterol; TyG, triglycerides-glucose index; CTI,high-sensitivity C-reactive protein-triglyceride glucose index; SBP, systolic blood pressure; DBP, diastolic blood pressure; *P* ≤ 0.05 was considered statistically significant.

### 3.2 The effect of CTI on CKD

**Figure 1:**
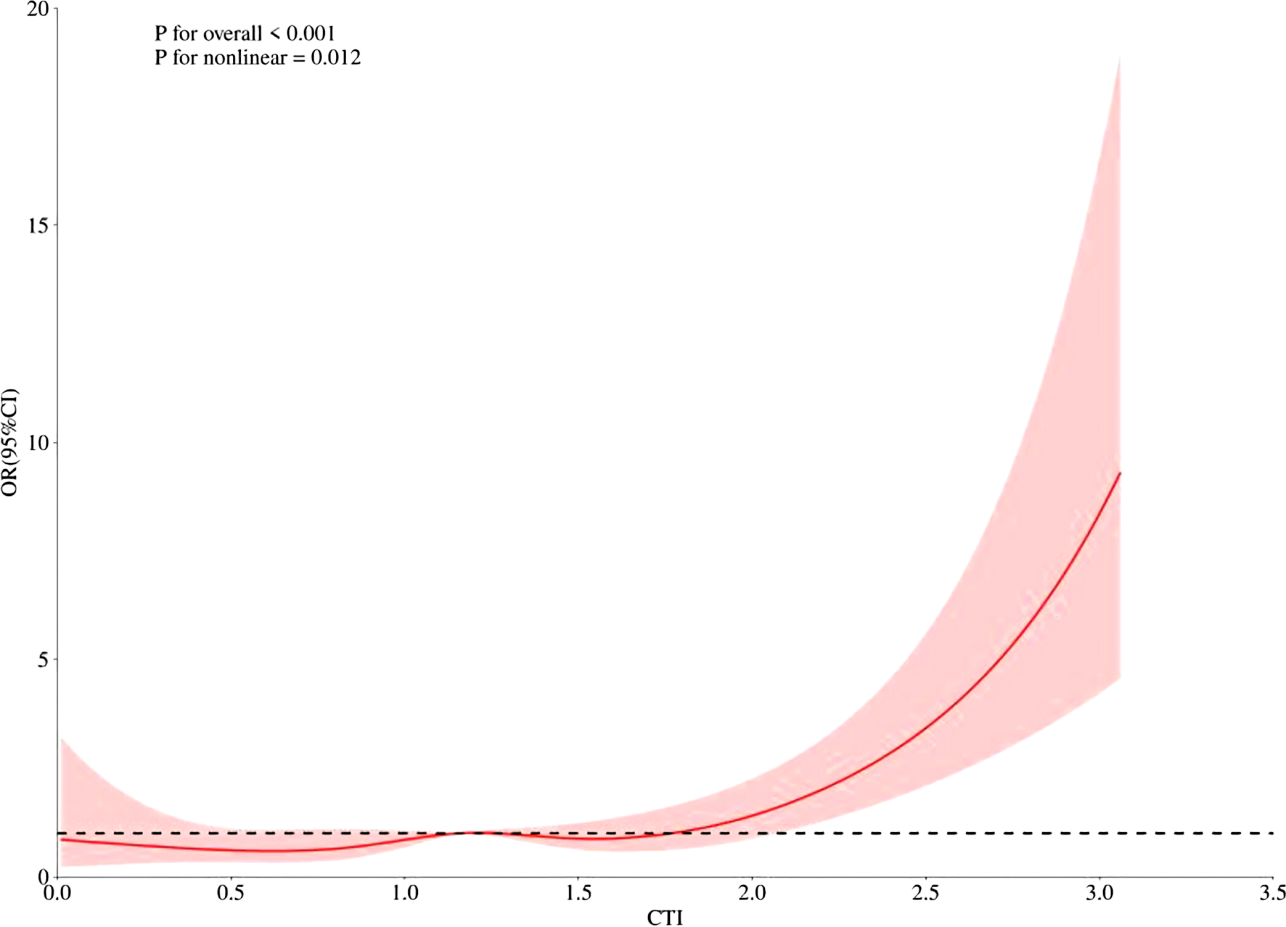
The RCS regression between the CTI and the risk of CKD.

The results of the logistic regression analyses were presented in **Table 2**. A higher CTI was significantly associated with an increased risk of CKD. This positive association remained consistent across all adjusted models, with a fully adjusted OR of 2.25 (95% CI: 1.66–3.06) in Model III. When CTI was categorized into quartiles, participants in the highest quartile (Q4) exhibited significantly higher odds of CKD compared to those in the lowest quartile (Q1) in the fully adjusted model (OR = 2.17, 95% CI: 1.25–3.76). A significant dose-response relationship was observed, as indicated by a positive trend across increasing CTI quartiles (*P* for trend = 0.001). Specifically, the OR for Q2 and Q3 in Model III were 1.05 (95% CI: 0.58–1.89) and 1.15 (95% CI: 0.64–2.05), respectively; although these estimates were not statistically significant, they consistently pointed toward an increased risk. The RCS regression model showed a non-linearly increasing relationship between the CTI and CKD (*P*-Nonlinear =0.012) (**Figure 1**).

**Table 2:** Logistic regression analysis for the association between CTI and CKD.

Variables	OR (95%CI)		
	Model I	Model II	Model III
CTI	3.08 (2.36, 4.02)	2.49 (1.84, 3.39)	2.25 (1.66, 3.06)
CTI (quartiles)			
Q1	1.00(ref)	1.00(ref)	1.00(ref)
Q2	1.60 (0.90, 2.86)	1.08 (0.60, 1.94)	1.05 (0.58, 1.89)
Q3	1.95 (1.12, 3.42)	1.18 (0.66, 2.10)	1.15 (0.64, 2.05)
Q4	4.21 (2.54, 6.98)	2.36 (1.37, 4.08)	2.17 (1.25, 3.76)
*P* for trend	<0.001	<0.001	0.001

Model I: Crude model; Model II: Adjusted for age, gender, BMI; Model III: Further adjusted hypertension, diabetes, smoking, drinking.

### 3.3 The ROC curve for TyG, hs-CRP and CTI predicting CKD

**Figure 2:**
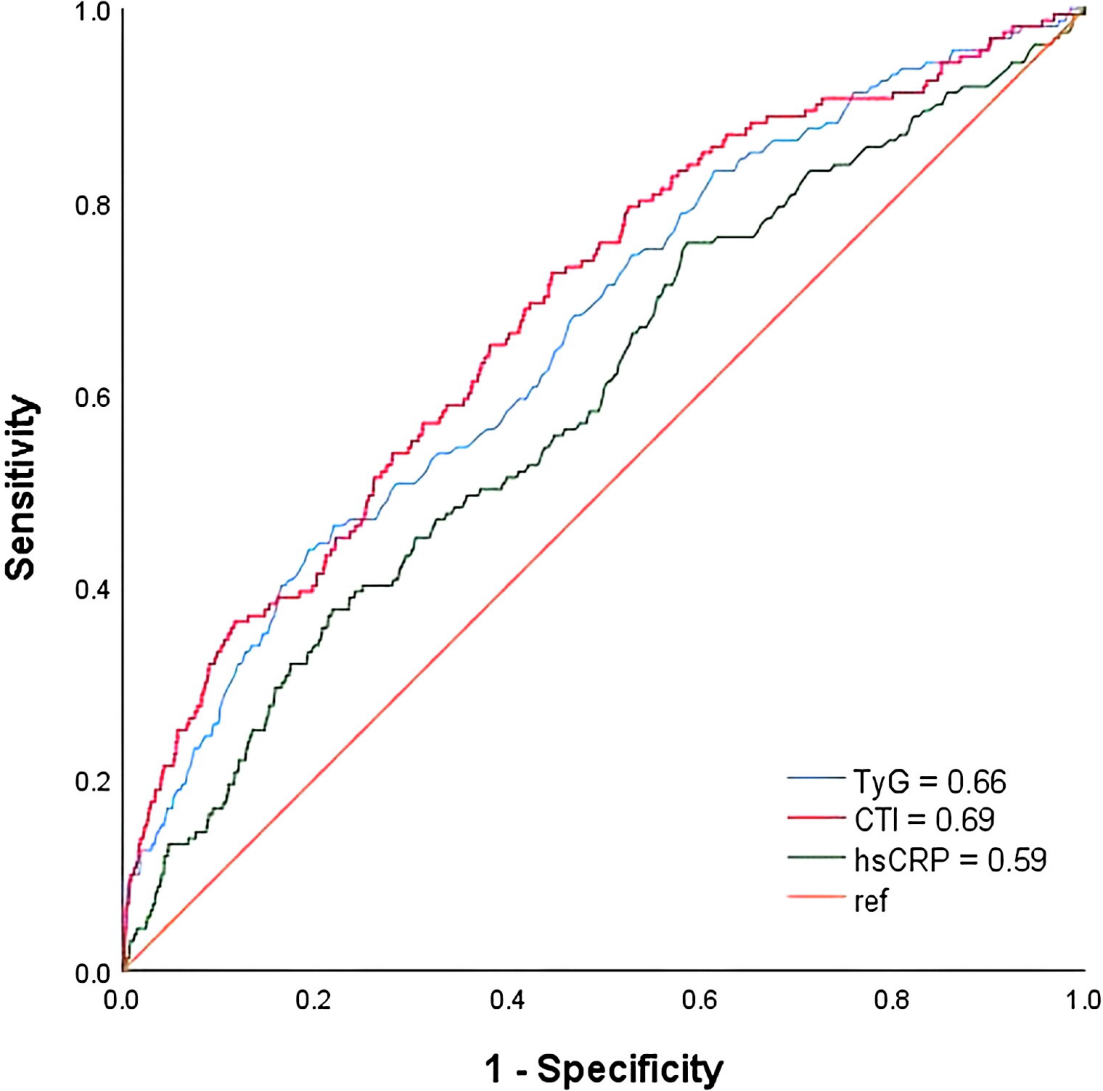
ROC curves for TyG, hs-CRP and CTI predicting CKD.

CTI (AUC 0.69, 95%CI 0.64-0.73) had the highest diagnostic efficacy for CKD, followed by TyG(AUC 0.66, 95%CI 0.62-0.70), and hs-CRP(AUC 0.59, 95%CI 0.55-0.64) (**Figure 2**).

### 3.4 Subgroup analysis

The results of the subgroup analysis were presented in **Table 3**.

**Table 3:** Association of CTI with the risk of CAD stratified by different factors.

Subgroup	OR (95%CI)	*P*
Gender		
Male	2.51 (1.74, 3.62)	<0.001
Female	1.70 (0.96, 3.01)	0.067
Age		
<60	2.91 (1.96, 4.30)	<0.001
≥60	1.40 (0.85, 2.30)	0.181
Smoking		
Yes	2.76 (1.55, 4.76)	<0.001
No	2.14 (1.47, 3.11)	<0.001
Drinking		
Yes	3.30 (2.04, 5.35)	<0.001
No	1.76 (1.18, 2.63)	0.005
Hypertension		
Yes	2.49 (1.63, 3.80)	<0.001
No	1.97 (1.25, 3.11)	0.004
Diabetes		
Yes	1.93 (1.09, 3.40)	0.024
No	2.46 (1.703, 3.56)	<0.001

Subgroup analysis revealed that the positive association between CTI and CKD remained consistent across the vast majority of populations. The strength of the association exhibited significant heterogeneity across different subgroups: it was particularly pronounced in males (OR = 2.51, 95% CI: 1.74–3.62), individuals aged <60 years (OR = 2.91, 95% CI: 1.96–4.30), and those with a history of alcohol consumption (OR = 3.30, 95% CI: 2.04–5.35). In contrast, the association did not reach statistical significance in females (OR = 1.70, 95% CI: 0.96–3.01) or individuals aged ≥60 years (OR = 1.40, 95% CI: 0.85–2.30). Furthermore, among participants without a history of diabetes (OR = 2.46), the effect size of CTI was slightly higher than that among those with a history of diabetes (OR = 1.93).

## 4 Discussion

In this large-scale cross-sectional analysis of 10–287 health examination participants, we developed and evaluated a novel composite biomarker, the CTI, which integrates hs-CRP and the TyG index to quantify the combined burden of systemic inflammation and insulin resistance. A higher CTI level was independently and strongly associated with an increased prevalence of CKD, even after comprehensive adjustment for traditional cardiometabolic risk factors. This association was robust, demonstrating a clear dose-response relationship across CTI quartiles and persisting after rigorous adjustment for a comprehensive panel of traditional confounders, including age, blood pressure, and diabetes status. Notably, the relationship was nonlinear, with the risk of CKD accelerating beyond a certain threshold of CTI, suggesting a potential critical point of metabolic-inflammatory toxicity. The diagnostic utility of CTI (AUC = 0.69) surpassed that of its individual components, hs-CRP (AUC = 0.59) and the TyG index (AUC = 0.66), underscoring the additive value of the composite metric. Furthermore, we identified significant heterogeneity in the strength of this association across population subgroups; it was markedly more pronounced in males, individuals younger than 60 years, and those with a history of alcohol consumption.

The association between CTI and CKD is strongly supported by and provides epidemiological reinforcement for the “metabolic inflammation” paradigm in CKD pathogenesis. This model posits that insulin resistance and chronic low-grade inflammation are not merely concurrent phenomena but are mechanistically interlinked drivers of renal injury (22, 23). Our finding that CTI, a multiplicative term of hs-CRP and TyG, was a stronger correlate of CKD than either component alone offers compelling evidence for synergistic, rather than simply additive, interaction. Insulin resistance, proxied by the TyG index, inflicts renal damage through multiple pathways. It induces glomerular hyperfiltration and hypertension, activates the intrarenal renin-angiotensin-aldosterone system (RAAS), and promotes ectopic lipid accumulation in renal cells (lipotoxicity), leading to podocyte injury and tubular dysfunction (24–26). Concurrently, chronic inflammation, indicated by elevated hs-CRP, creates a deleterious microenvironment through the sustained release of cytokines (e.g., IL-1β, IL-6, TNF-α) (27). These molecules promote vascular endothelial dysfunction, recruit inflammatory cells, stimulate oxidative stress, and directly activate pro-fibrotic pathways, ultimately leading to glomerulosclerosis and tubulointerstitial fibrosis (28, 29). Critically, these pathways feed into each other: insulin resistance can stimulate inflammatory signaling (e.g., via NF-κB), while inflammatory cytokines can directly interfere with insulin signaling, creating a vicious, self-perpetuating cycle that the CTI appears to capture effectively (30–32).

The nonlinear relationship revealed by the RCS analysis was a significant and clinically relevant finding. It implies the existence of a physiological threshold or tipping point in metabolic-inflammatory burden. Below this point, compensatory mechanisms may mitigate renal damage, but once exceeded, decompensation occurs, leading to a disproportionate acceleration in CKD risk. This nonlinearity may explain why studies using linear models or focusing on populations with lower baseline risk sometimes report attenuated associations. It also suggests that interventions aimed at reducing CTI might yield disproportionate renal benefits once this threshold is crossed.

The observed subgroup heterogeneity provides deeper insights into population-specific risk. The stronger association in males may be attributed to sex-specific differences in body composition, hormonal profiles, or a higher prevalence of lifestyle risk factors traditionally linked to metabolic inflammation. The attenuated association in females may be related to several factors. Estrogen is known to have modulatory effects on both systemic inflammation and insulin sensitivity, potentially rendering the CTI, which is heavily weighted towards these pathways, a less sensitive marker in pre-menopausal women. Furthermore, the lower absolute risk and potentially different etiological pathways for CKD in females in our cohort might contribute to reduced statistical power to detect a significant association within this subgroup. The markedly stronger effect in individuals aged <60 years was particularly noteworthy. It suggests that CTI is a potent marker of “premature” or accelerated renal aging driven by modifiable metabolic and inflammatory factors. The lack of a significant association in individuals aged ≥60 years likely reflects the increasing etiological heterogeneity of CKD with advancing age. While ‘metabolic inflammation’ may be a primary driver in younger and middle-aged adults, in older populations, other non-metabolic, age-related pathologies become predominant. These include hypertensive nephrosclerosis, ischemic nephropathy, senescent nephron loss, and medication-related interstitial diseases. This etiological shift effectively ‘dilutes’ the specific signal captured by the CTI, reducing its discriminatory power in an older, clinically complex population. The notably strong association observed among individuals reporting alcohol consumption warrants cautious interpretation. While this finding suggests a potential interaction between metabolic-inflammatory status and alcohol use, it cannot distinguish between causality, effect modification, or residual confounding. Alcohol consumption patterns and associated lifestyle factors were not fully characterized and may confound this relationship. It is plausible that alcohol, particularly in certain patterns, exacerbates metabolic inflammation, but it is equally plausible that this subgroup has other unmeasured risk profiles. Therefore, this observation should be considered hypothesis-generating, highlighting an important avenue for future research that includes detailed assessment of drinking patterns and comprehensive lifestyle data.

Our findings align with and extend the growing body of literature on metabolic and inflammatory markers in CKD. Numerous studies had independently associated the TyG index with reduced eGFR and incident CKD (33–35), and several studies had confirmed hs-CRP as an independent risk factor for CKD development and progression (12, 36, 37). However, by integrating them multiplicatively into the CTI, we demonstrate superior discriminative performance. This aligns with the evolving understanding that composite indices often outperform single biomarkers for complex diseases. For instance, similar integrative approaches, such as the Systemic Immune-Inflammation Index (SII) or combinations of obesity and inflammation markers, have shown promise in predicting cardiorenal outcomes (18, 38). Our study contributes to this trend by proposing a specifically metabolism-focused inflammatory index.

The CTI holds several potential translational implications. First, as a low-cost, calculable index derived from routine blood tests (hs-CRP, triglycerides, glucose), it could be seamlessly integrated into health check-up reports or electronic health records to flag individuals at high risk for subclinical CKD. The nonlinear risk threshold identified could inform future studies to establish clinically actionable cut-off values for targeted screening. Second, the superior performance of CTI advocates for a paradigm shift in risk assessment from a single-biomarker approach to a multidimensional profiling strategy. Clinicians should be encouraged to consider both inflammatory and metabolic axes simultaneously when evaluating a patient’s renal health, particularly in younger and middle-aged adults. Third, the subgroup findings advocate for precision prevention. Screening efforts utilizing CTI could be prioritized for younger and middle-aged men undergoing health examinations, as they may derive the greatest benefit from early detection. For individuals with a high CTI, especially those below the age of 60, aggressive management of modifiable factors—through lifestyle intervention could be strongly justified to preserve renal function.

The key strengths of this study include the large, well-phenotyped sample from a real-world health examination setting, enhancing the generalizability of our findings to similar asymptomatic populations. The comprehensive adjustment for confounders and the extensive sensitivity and subgroup analyses bolster the robustness and credibility of our conclusions. However, several limitations must be acknowledged. First, and most critically, the cross-sectional design precludes any causal inference regarding the direction of the observed association. While we hypothesize that elevated metabolic-inflammatory load (CTI) contributes to kidney damage, reverse causality remains a plausible alternative. Impaired kidney function can itself exacerbate insulin resistance, dyslipidemia, and chronic inflammation through mechanisms such as uremic toxin accumulation, altered adipokine metabolism, and chronic low-grade oxidative stress (39, 40). Therefore, our findings should be interpreted as identifying a strong association between CTI and CKD, rather than establishing causation. Future prospective cohort studies are essential to determine the temporal relationship. Second, our CKD definition was based on a single measurement of eGFR < 60 mL/min/1.73 m² rather than the gold-standard ACR. This does not fulfill the KDIGO guideline requirement for confirmed persistence over at least three months, potentially leading to the inclusion of individuals with acute or transient kidney function changes. This misclassification, if non-differential, would likely attenuate the observed association toward the null, meaning our effect estimates might be conservative. Third, despite extensive adjustment, residual confounding by unmeasured or imperfectly measured factors (e.g., dietary patterns, physical activity intensity, medication use like statins or anti-inflammatories, or genetic predisposition) cannot be excluded. Fourth, the study population was drawn from a single-center health examination cohort, which may limit the generalizability of our findings to other ethnicities, socioeconomic groups, or healthcare settings. Moreover, the final cohort represents a complete-case sample after applying exclusion criteria. This may introduce selection bias, as health check-up participants may differ systematically from the general population, and individuals with missing data may have different risk profiles. This likely affects the generalizability of prevalence estimates but is less likely to invalidate the internal associations observed.

In conclusion, this study demonstrates that the CTI, a novel integrative index of metabolic inflammation, is independently, nonlinearly, and strongly associated with the CKD in a general adult population. Its performance exceeds that of its individual components, validating the concept that assessing the synergistic burden of insulin resistance and inflammation provides a superior risk signal. The association is particularly strong in men and younger individuals, highlighting target groups for focused screening.

## Data Availability

The datasets, code book, and analytic code described in the article are not readily available because the research subject requires data confidentiality. Requests to access the datasets should be directed to 13662052236@163.com.
